# Reduction of Circulating Endothelial Progenitor Cell Level Is Associated with Contrast-Induced Nephropathy in Patients Undergoing Percutaneous Coronary and Peripheral Interventions

**DOI:** 10.1371/journal.pone.0089942

**Published:** 2014-03-19

**Authors:** Chia-Hung Chiang, Po-Hsun Huang, Chun-Chih Chiu, Chien-Yi Hsu, Hsin-Bang Leu, Chin-Chou Huang, Jaw-Wen Chen, Shing-Jong Lin

**Affiliations:** 1 Division of Cardiology, Department of Medicine, Taipei Veterans General Hospital, Hsinchu Branch, Hsinchu, Taiwan; 2 Division of Cardiology, Taipei Veterans General Hospital, Taipei, Taiwan; 3 Department of Medical Research and Education, Taipei Veterans General Hospital, Taipei, Taiwan; 4 Healthcare and Management Center, Taipei Veterans General Hospital, Taipei, Taiwan; 5 Institute of Clinical Medicine, National Yang-Ming University, Taipei, Taiwan; 6 Cardiovascular Research Center, National Yang-Ming University, Taipei, Taiwan; 7 Institute and Department of Pharmacology, National Yang-Ming University, Taipei, Taiwan; French Blood Institute, France

## Abstract

**Objectives:**

Reduced number and impaired function of circulating endothelial progenitor cells (EPCs) in patients with chronic kidney disease have been reported. However, there is little data about the association between circulating EPC levels and risk of contrast-induced nephropathy (CIN). The aim of this study was to investigate the relationship between circulating EPCs and CIN in patients after angiography.

**Methods and Results:**

A total of 77 consecutive patients undergoing elective percutaneous coronary intervention (PCI) and percutaneous transluminal angioplasty (PTA) were enrolled. Flow cytometry with quantification of EPC markers (defined as CD34^+^, CD34^+^KDR^+^, and CD34^+^KDR^+^CD133^+^) in peripheral blood samples was used to assess EPC number before the procedure. CIN was defined as an absolute increase ≧0.5 mg/dl or a relative increase ≧25% in the serum creatinine level at 48 hours after the procedure. Eighteen (24%) of the study subjects developed CIN. Circulating EPC levels were significantly lower in patients who developed CIN than in those without CIN (CD34^+^KDR^+^, 4.11±2.59 vs. 9.25±6.30 cells/10^5^ events, P<0.001). The incidence of CIN was significantly greater in patients in the lowest EPC tertile (CD34^+^KDR^+^; from lowest to highest, 52%, 15%, and 4%, P<0.001). Using univariate logistic regression, circulating EPC number (CD34^+^KDR^+^) was a significant negative predictor for development of CIN (odds ratio 0.69, 95% CI 0.54–0.87, P = 0.002). Over a two-year follow-up, patients with CIN had a higher incidence of major adverse cardiovascular events including myocardial infarction, stroke, revascularization of treated vessels, and death (66.7% vs. 25.4%, P = 0.004) than did patients without CIN.

**Conclusions:**

Decreased EPC level is associated with a greater risk of CIN, which may explain part of the pathophysiology of CIN and the poor prognosis in CIN patients.

## Introduction

Contrast-induced nephropathy (CIN) remains a serious clinical problem in the use of iodinated contrast media [1], [2]. Increasing use of contrast media in interventional procedures has led to a parallel increase in the incidence of CIN, despite the use of newer and less nephrotoxic contrast agents in high-risk patients in recent years. The reported incidence of CIN varies widely across the literature [1], [3]. Its development has been associated with increased in-hospital and long-term morbidity and mortality, prolonged hospitalization, and long-term renal impairment [4]. Proposed pathophysiologic mechanisms through which contrast administration may potentiate renal injury include oxidative stress, free radical damage, and endothelial dysfunction [5], [6]. However, the actual pathogenesis of CIN and the pathophysiologic mechanisms underlying the evolution from CIN to atherosclerosis and cardiovascular events remain to be determined.

Vascular endothelium is a highly active organ that affects vascular tone, smooth muscle cell proliferation, monocyte adhesion, and platelet aggregation [7], [8]. Endothelial dysfunction plays a critical role in the clinical manifestations of established atherosclerotic lesions. Clinical studies have demonstrated that endothelial dysfunction is present in the early stages of renal insufficiency, and that it is associated with a greater decline in renal function [9], [10]. Recent insight suggests that the injured endothelial monolayer is regenerated by circulating bone marrow derived-endothelial progenitor cells (EPCs), and levels of circulating EPCs reflect endothelial repair capacity. An altered status of circulating EPCs represents a marker of endothelial dysfunction and vascular health, and the level of circulating EPCs could be used as a surrogate index of cumulative cardiovascular risk [11], [12]. A reduced number of circulating EPCs independently predicts atherosclerotic disease progression and future cardiovascular events [13]. Furthermore, previous reports have indicated reduced number and impaired function of EPCs in chronic renal insufficiency [9]. However, there is currently little data about the association between circulating EPC levels and risk of CIN. To clarify this issue, we tested the hypothesis that decreased circulating EPC levels may be associated with increased risk of CIN and subsequent major cardiovascular events in patients undergoing cardiovascular interventional procedures.

## Methods

### Study Participants

We initially screened a total of 311 consecutive patients who were admitted to the ward at the Division of Cardiology, Taipei-Veterans General Hospital between October 2009 and January 2010. Patients, who were older than 18 years of age, with normal to subnormal GFR, and scheduled for elective cardiovascular procedures including percutaneous coronary intervention (PCI) and percutaneous transluminal angioplasty (PTA), were eligible for this study. Exclusion criteria were as follows: hemodynamically significant valvular disorders, uncontrolled hypertension, baseline serum creatinine levels of more than 7 mg/dL, preexisting dialysis, autoimmune disease, chronic or acute infectious disease, emergency catheterization, recent exposure to radiographic contrast within 10 days, medication with non-steroidal anti-inflammatory drugs or metformin up to 7 days before entering the study, anemia (hemoglobin level <12 g/dl), overt congestive heart failure, recent acute kidney injury, having another planned contrast-enhanced procedure within the following 72 hours, and allergy to radiographic contrast. On the basis of these screening criteria, we enrolled 77 patients in the current study (48 patients receiving PCI, 29 patients receiving PTA). Medical history, including information about conventional cardiovascular risk factors (smoking, hypertension, diabetes mellitus, hyperlipidemia, peripheral artery disease, coronary artery disease, and chronic kidney disease), previous cardiovascular events (myocardial infarction and cerebrovascular disease), and current drug treatment was obtained during a personal interview and from medical files. This study was approved by the Taipei Veterans General Hospital research ethics committee. All patients gave written informed consent and research was conducted according to the principles expressed in the *Declaration of Helsinki*.

### Study Treatment and Cardiovascular Procedures

All patients received a periprocedural intravenous infusion (volume expansion) of 1 ml/kg/h with 0.45% saline for 24 hours (12 hours before and 12 hours after exposure to contrast medium). On the day before the procedure, the estimated glomerular filtration rate (eGFR) was assessed using the modified formula of Levey *et al* [14]. Chronic kidney disease was defined as an eGFR <60 ml/min/1.73 m^2^, based on the recommendations of the National Kidney Foundation [15]. CIN was defined as an absolute increase ≧0.5 mg/dl or a relative increase ≧25% in the serum creatinine level within 48 hours after the procedure.

The performance of angiography, PCI and PTA was left to the discretion of the cardiologists responsible for the patient and the interventional cardiologist on the basis of current guidelines. Cardiologists performing cardiovascular procedures were blinded to EPC levels of study subjects. A nonionic iso-osmolar contrast agent (Iodixanol, Visipaque) was used in all patients. During hospitalization, medications were changed as required by the clinical situation. All study subjects also underwent a complete echocardiographic study, including tissue Doppler imaging, upon enrollment in this study.

### Laboratory Investigations

Venous blood was drawn in the morning after overnight fasting. Plasma liver function tests and other biochemical blood measurements, including assessments of fasting blood glucose, uric acid, creatinine, total cholesterol, high-density lipoprotein cholesterol (HDL-C), and triglyceride levels were performed by standard laboratory procedures. The high-sensitivity C-reactive protein (hsCRP) levels in plasma were assessed using latex-enhanced immunonephelometric assay (Dade Behring, Marburg, Germany). Serum levels of matrix metalloproteinase-2 (MMP-2) and matrix metalloproteinase-9 (MMP-9) were determined using commercially available enzyme-linked immunoassays. Study subjects were also tested for Cystatin C and nitric oxide (NO) levels. Total NO assay was performed by spectrophotometry at 540 nm using an NO assay kit according to the manufacturer’s instructions. The assay was based on nitrate and nitrite determinations.

### Assay of Circulating EPCs

**Figure 1:**
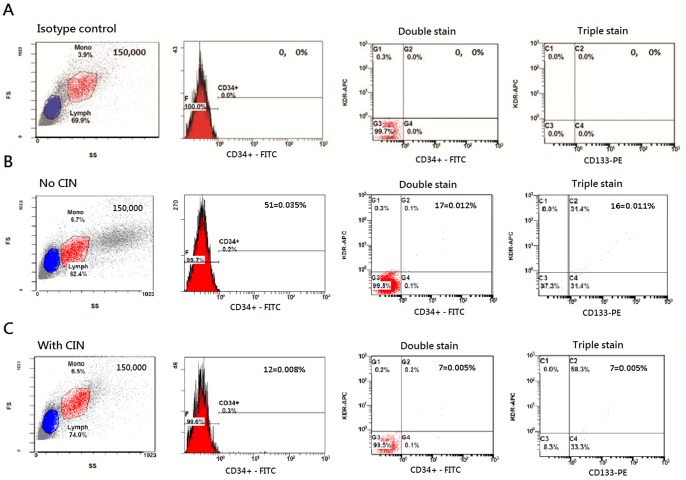
Representative flow cytometry analysis for quantifying the number of circulating endothelial progenitor cells in study subjects: (A) isotype control, (B) patients without CIN (C) patients with CIN. The numbers of circulating EPCs were gated with monocytes and defined as CD34^+^CD45^low^, CD34^+^KDR^+^CD45^low^, and CD34^+^KDR^+^CD133^+^CD45^low^. CIN, contrast-induced nephropathy.

Assessment of the circulating EPCs by flow cytometry was performed by researchers masked to the clinical data [16]. A volume of 1000 μL of peripheral blood was incubated for 30 min in the dark with monoclonal antibodies against human kinase insert domain receptor (KDR) (R&D, Minneapolis, MN, USA), followed by allophycocyanin (APC)-conjugated secondary antibody, with the fluorescein isothiocyanate (FITC)-labeled monoclonal antibodies against human CD45 (Becton Dickinson, Franklin Lakes, NJ, USA), with the phycoerythrin (PE)-conjugated monoclonal antibody against human CD133 (Miltenyi Biotec, Germany), and with FITC-conjugated monoclonal antibodies against human CD34 (Becton Dickinson Pharmingen, USA). After incubation, the cells were lysed, washed with phosphate-buffered saline (PBS), and fixed in 2% paraformaldehyde before analysis. Each analysis included 100,000 events. The numbers of circulating EPCs were gated with monocytes and defined as CD34^+^, CD34^+^KDR^+^, and CD34^+^KDR^+^CD133^+^ (**Figure 1**). To assess the reproducibility of EPC measurements, circulating EPCs were measured from 2 separate blood samples in 10 subjects, and there was a strong correlation between the 2 measurements (r = 0.90, P<0.001).

### Assessment of Major Cardiovascular Events

All subjects included in this study were followed up for a maximum of 2 years or until death. The primary endpoint of the current study was the development of major adverse cardiovascular events (MACE), including the composite of all-cause death, cardiovascular death, nonfatal myocardial infarction, stroke, and revascularization of treated vessels. Cardiovascular death was defined as death from cardiac causes, cardiac arrest, myocardial infarction, and stroke. Stroke was diagnosed based on the presence of a neurologic deficit confirmed by computed tomography or magnetic resonance imaging. No study subjects dropped out of the study, and all occurrences of adverse events were recorded. Outcome data were collected by serial contact with the patients or their families until March 31, 2012.

### Statistical Analysis

Data were expressed as the mean ± standard deviation (SD) or median with interquartile range for numeric variables and as the number (percent) for categorical variables. Comparisons of continuous variables between 2 or more groups were performed by Student’s *t* test and ANOVA, respectively; post-hoc comparisons were performed by Tukey’s honest significant difference test. Subgroup comparisons of categorical variables were assessed by the chi-squared test or Fisher’s exact test. To examine the effects of various factors on development of CIN, the following factors were considered as variables for univariate and multivariate logistic regression analyses: EPC number (CD34^+^KDR^+^), age, gender, hypertension, diabetes, chronic kidney disease, heart failure, and contrast volume. To assess the risk of developing MACE during the 2 year follow-up period, the Kaplan-Meier method was employed for patients stratified by EPC levels. Data were analyzed using SPSS software (version 17, SPSS, Chicago, Illinois, USA). A P value of <0.05 was considered to indicate statistical significance.

## Results

### Clinical and Laboratory Data

A total of 77 subjects (mean age: 69±15 years; male subjects, 63 [82%]) were enrolled in the study. Of the 77 study subjects, 48 received PCI and 29 received PTA. Eighteen patients developed CIN after the procedures, giving an overall CIN incidence of 24% in the current study, with three of the CIN patients requiring dialysis. All patients were divided into two groups; those who developed CIN and those who did not. **Table 1** summarizes the demographic and clinical characteristics of study subjects. Baseline demographics and characteristics were comparable between these two groups. Baseline metabolic profiles and medication use are presented in **Table 2**. There was no significant difference between the two groups in regard to the baseline creatinine levels, metabolic profiles, and medication use. No significant differences were noted in angiographic and procedural characteristics of study subjects undergoing PCI and PTA between the two groups, except that CIN patients had higher post-procedural creatinine levels than those without CIN (CIN vs. non-CIN, 1.9±1.4 vs. 1.1±0.3, P = 0.019; **Table 3**).

**Table 1:** Baseline characteristics of study subjects with and without contrast-induced nephropathy (CIN).

	No CIN	With CIN	P value
	n = 59	n = 18	
Age (years)	67.3±15.3	72.6±13.8	0.190
Men, n (%)	48 (81.4%)	15 (83.3%)	0.849
Hypertension, n (%)	43 (72.9%)	16 (88.9%)	0.213
Diabetes mellitus, n (%)	31 (52.5%)	11 (61.1%)	0.596
Coronary artery disease, n (%)	52 (88%)	17 (94%)	0.672
Peripheral artery disease, n (%)	4 (14.8%)	14 (31.1%)	0.132
Chronic kidney disease, n (%)	36 (61.0%)	10 (55.6%)	0.785
Hyperlipidemia, n (%)	36 (61.0%)	10 (55.6%)	0.785
Current smoker, n (%)	28 (47.5%)	9 (50.0%)	0.851
Previous myocardial infarction, n (%)	22 (37.3)	9 (50.0%)	0.414
Previous cerebrovascular disease, n (%)	11 (18.6%)	3 (16.7%)	0.849
Heart failure, n (%)	15 (25.4%)	5 (27.8%)	0.842
Atrial fibrillation, n (%)	14 (23.7%)	3 (16.7%)	0.748

Values are mean ± standard deviation (SD) or number (%).

CAD, coronary artery disease; PCI: percutaneous coronary intervention.

**Table 2:** Baseline metabolic profiles and medications of subjects with/without contrast-induced nephropathy (CIN).

	No CIN	With CIN	
	n = 59	n = 18	P value
Cholesterol (mg/dL)	172±46	160±23	0.173
LDL-C (mg/dL)	109±39	96±26	0.252
HDL-C (mg/dL)	45±21	37±11	0.171
Triglyceride (mg/dL)	117±70	111±70	0.754
Creatinine (mg/dL)	1.1±0.4	1.4±1.2	0.365
eGFR, ml/min/1.73 m^2^	68±27	65±32	0.692
ALT (U/L)	33±35	30±33	0.731
Fasting glucose (mg/dL)	143±67	152±62	0.632
Body mass index (kg/m^2^)	26.0±4.2	25.0±4.1	0.360
Medication, n (%)			
Aspirin	52 (88.1%)	14 (77.8%)	0.272
Clopidogrel	42 (71.2%)	10 (55.6%)	0.256
Cilostazol	21 (35.6%)	7 (38.9%)	0.787
ACEI	9 (15.3%)	4 (22.2%)	0.488
ARB	23 (39.0%)	5 (27.8%)	0.576
CCB	25 (42.3%)	8 (44.4%)	0.876
Beta blocker	19 (32.2%)	8 (44.4%)	0.402
Diuretics	15 (25.4%)	4 (22.2%)	0.783
Insulin	5 (8.5%)	3 (16.7%)	0.381
Statins	31(52.5%)	6 (33.3%)	0.185
Nitrates	31 (52.5%)	7 (38.9%)	0.421

Values are presented as mean ± standard deviation (SD) or number (%).

LDL-C: low-density lipoprotein cholesterol; HDL-C: high-density lipoprotein cholesterol; ALT: alanine aminotransferase; γGT: gamma-glutamyl-transferase; ACEI: angiotensin-converting enzyme inhibitor; ARB: angiotensin receptor blocker; CCB: calcium channel blocker.

**Table 3:** Angiographic and procedural characteristics of study subjects undergoing coronary artery intervention (PCI) and percutaneous transluminal angioplasty (PTA).

	No CIN	With CIN	P value
	n = 59	n = 18	
Undergoing PCI, n (%)	36 (61.0)	12(66.7)	0.784
Undergoing PTA, n (%)	23 (39.0)	6 (20.7)	0.784
Pre-procedural creatinine (mg/dL)	1.1±0.4	1.4±1.2	0.365
*Post-procedural creatinine (mg/dL)	1.1±0.3	1.9±1.4	0.019
SYNTAX score in CAD patients	16.6±11.5	18.0±9.9	0.648
CAD with left main disease, n (%)	8 (13.6)	5 (27.8)	0.169
Treated coronary artery, n (%)			
Left anterior descending	24 (40.7)	5 (27.8)	0.410
Left circumflex	8 (13.6)	3 (16.7)	0.712
Right coronary	14 (23.7)	5 (27.8)	0.760
Complexity of CAD, n (%)			
Multivessel disease	22 (37.3)	9 (50.0)	0.414
Bifurcation lesion	11 (18.6)	5 (27.8)	0.508
Chronic total occlusion	5 (8.4)	2 (11.1)	0.663
Number of treated segments per CAD patient	1.6±1.5	1.5±1.7	0.824
Number of stent deployments per CAD patient	1.3±1.6	1.4±2.2	0.796
Deployment of coronary BMS, n (%)	14 (23.7)	3 (16.7)	0.748
Deployment of coronary DES, n (%)	24 (40.7)	6 (33.3)	0.783
ABI in PAD patients	0.55±0.31	0.61±0.21	0.571
Treated peripheral arteries, n (%)			
Common iliac artery	2 (3.4)	1 (5.6)	0.556
Superficial femoral artery	17 (28.8)	4 (22.2)	0.765
Below -knee arteries	16 (27.1)	4 (22.2)	0.768
Contrast volume (mL)	210±136	242±136	0.190

Values are mean ± standard deviation (SD) or number (%).

CIN, contrast-induced nephropathy; ABI, ankle-brachial index; CAD, coronary artery disease; BMS, bare-metal stent; DES, drug-eluting stent.

*Post-procedural creatinine: 48 hours after the procedures.

### Circulating EPC Levels and Other Biomarkers

**Figure 2:**
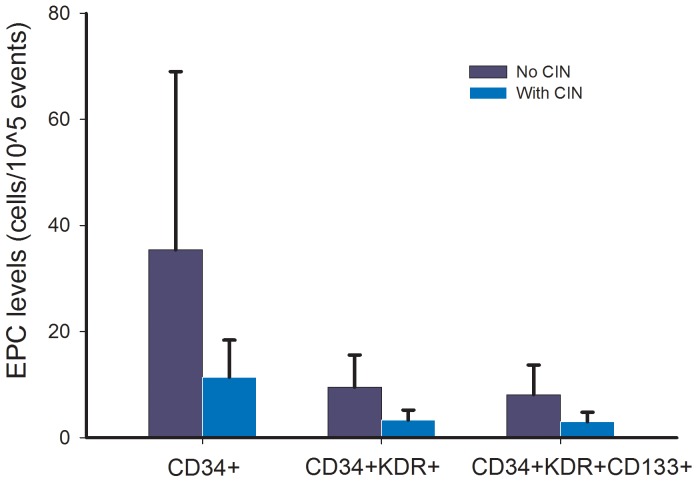
EPC levels (cells/10^5^ events) in patients with and without development of contrast-induced nephropathy (CIN) (values presented as means ± standard deviation).

As shown in **Table 4** and **Figure 2**, there were decreased circulating CD34^+^ cells in CIN patients compared to non-CIN patients (CIN vs. non-CIN, CD34^+^: 0.011±0.007 vs. 0.035±0.033%, P = 0.004). Additionally, the EPC markers defined as CD34^+^KDR^+^ and CD34^+^KDR^+^CD133^+^ were significantly decreased in CIN patients compared to non-CIN patients (CIN vs. non-CIN, CD34^+^KDR^+^: 0.003±0.001 vs. 0.012±0.010%, P<0.001; CD34^+^KDR^+^CD133^+^: 0.003±0.002 vs. 0.010±0.010%, P<0.001). Furthermore, CIN patients had significantly enhanced Cystatin C levels (CIN vs. non-CIN, 1.4±0.8 vs. 0.9±0.3 mg/dl, P = 0.046) and reduced NO levels (CIN vs. non-CIN, 33±24 vs. 51±29 μmol/l, P = 0.031). However, no significant difference was noted in plasma levels of hsCRP between the two groups.

**Table 4:** Circulating endothelial progenitor cell (EPC) levels and other markers.

	No CIN	With CIN	P value
	n = 59	n = 18	
EPC levels (%)			
CD34^+^	0.035±0.033	0.011±0.007	0.004
CD34^+^KDR^+^	0.012±0.010	0.003±0.001	0.001
CD34^+^KDR^+^CD133^+^	0.010±0.010	0.003±0.002	<0.001
EPC levels (cells/10^5^ events)			
CD34^+^	35.5±33.6	11.4±7.0	0.004
CD34^+^KDR^+^	9.5±6.1	3.3±1.9	<0.001
CD34^+^KDR^+^CD133^+^	8.1±5.6	3.1±1.8	<0.001
hsCRP (mg/L)	0.4 (0.2–1.1)	0.9 (0.2–3.3)	0.191
Nitric oxide (μmol/L)	51±29	33±24	0.031
Cystatin C (mg/dL)	0.9±0.3	1.4±0.8	0.046
MMP-2 (ng/mL)	151±45	159±45	0.545
MMP-9 (ng/mL)	55±37	44±19	0.314

Values are mean ± SD or median (interquartile range).

CIN, contrast-induced nephropathy; hsCRP: high-sensitivity C-reactive protein; MMP: matrix metalloproteinase.

### Independent Correlates of Development of CIN

In order to identify the independent predictors for development of CIN, univariate and multivariate logistic regression analyses were performed. As shown in **Table 5**, in univariate analysis, EPC number (CD34^+^KDR^+^, cells/10^5^ events) was noted to be a significant negative predictor for development of CIN (crude odds ratio [95% CI]: 0.49 [0.34–0.72], P<0.001). In multivariate analysis, regardless of adjusting for other confounders like age, gender (male), hypertension, diabetes, chronic kidney disease, heart failure, or contrast volume, EPC number was still inversely associated with risk of CIN (P<0.001).

**Table 5:** Association between endothelial progenitor cell (EPC) levels and development of contrast-induced nephropathy (CIN).

EPCs (CD34^+^KDR^+^, cells/10^5^ events)	OR (95% CI)	P value
Univariate analysis	0.49 (0.34–0.72)	<0.001
Multivariate analysis		
Adjusted for age	0.48 (0.33–0.72)	<0.001
Adjusted for gender (male)	0.47 (0.31–0.71)	<0.001
Adjusted for hypertension	0.47 (0.32–0.71)	<0.001
Adjusted for diabetes	0.48 (0.33–0.71)	<0.001
Adjusted for chronic kidney disease	0.41 (0.26–0.67)	<0.001
Adjusted for heart failure	0.49 (0.33–0.72)	<0.001
Adjusted for contrast volume (mL)	0.40 (0.24–0.66)	<0.001

OR: odds ratio; CI: confidence interval.

### Incidence of Cardiovascular Events, All-cause Deaths, and CIN

**Table 6** illustrates the incidence of clinical outcomes in patients with and without CIN. Among the patients who developed CIN, the MACE rate was 67% compared with only 25% in patients who did not develop CIN during the maximum 2 years of follow-up period. Seven deaths occurred, with three of them considered to be cardiovascular deaths (two fatal myocardial infarction, one stroke-related death). Furthermore, CIN patients had significantly higher incidence of stroke and fatal/nonfatal myocardial infarction than patients without CIN.

**Table 6:** The incidence of clinical outcomes in patients with/without contrast-induced nephropathy (CIN).

	No CIN	With CIN	P value
Clinical outcomes, n (%)	n = 59	n = 18	
Stroke	3 (5.1)	4 (22)	0.048
Myocardial infarction	3 (5.1)	4 (22)	0.048
Revascularization of treated vessel	11 (18.6)	8 (44.4)	0.057
Cardiovascular death	1 (1.7)	2 (11.1)	0.135
All-cause death	4 (6.8)	3 (16.7)	0.202
Total number of MACE	15 (25.4)	12 (66.7)	0.004

MACE, major cardiovascular events including stroke, fatal/nonfatal myocardial infarction, revascularization of treated vessel, cardiovascular death, and all-cause death.

**Figure 3:**
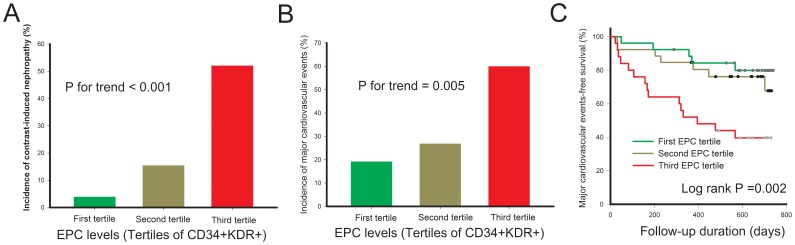
Association between tertiles of EPC level, and incidence of contrast-induced nephropathy (A), major cardiovascular events (MACE) (B), and MACE-free survival (C). First tertile: EPC (CD34^+^KDR^+^ ≧9 cells/10^5^ events); Second tertile: EPC (CD34^+^KDR^+^ = 5–8 cells/10^5^ events); Third tertile: EPC (CD34^+^KDR^+^ ≦ 4 cells/10^5^ events).

**Figure 4:**
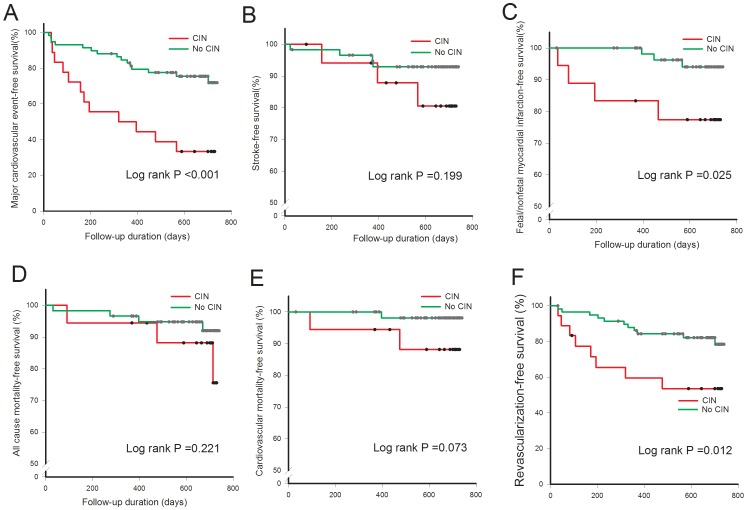
Association between contrast-induced nephropathy and major cardiovascular events (MACE), (A) MACE including stroke, fatal/nonfatal myocardial infarction, death and revascularization of treated vessels; (B) Stroke; (C) fatal/nonfatal myocardial infarction; (D) all-cause mortality; (E) cardiovascular death; (F) revascularization of treated vessels.

We further determined the relationship between circulating EPC level and incidence of CIN and future cardiovascular events after cardiovascular interventional procedures. All study subjects were divided into 3 groups according to circulating EPC levels (high-EPC level [first EPC tertile], CD34^+^KDR^+^ ≧9 cells/10^5^ events; intermediate-EPC level [second EPC tertile], CD34^+^KDR^+^ = 5–8 cells/10^5^ events; low-EPC level [third EPC tertile], CD34^+^KDR^+^ ≦ 4 cells/10^5^ events), and the incidence of CIN by tertiles of EPC levels is illustrated in **Figure 3A**. Patients with low-EPC levels had higher incidence of CIN compared to those with high-EPC levels and intermediate-EPC levels. Furthermore, as shown in **Figure 3B and 3C**, patients with low-EPC levels had more MACE during the follow-up period than the other two groups (High-EPC vs. Intermediate-EPC vs. Low-EPC: 19.2% vs. 26.9% vs. 60.0%, P = 0.005). Cumulative MACE-free survival in patients with and without CIN is shown in **Figure 4**. CIN patients had significantly lower cumulative event-free survival of MACE, fatal and nonfatal myocardial infarction, and revascularization of treated vessels compared to patients without CIN during the 2 year follow-up period.

## Discussion

This is the first study to show that decreased circulating EPC level is associated with a greater risk of CIN in patients undergoing percutaneous interventional procedures. Furthermore, patients with decreased circulating EPC number as well as CIN have increased cardiovascular events after percutaneous coronary or peripheral interventions. These findings suggest that reduced circulating EPC levels, reflecting attenuated endothelial repair capacity, may contribute to atherosclerotic disease progression and increased risk of cardiovascular events in patients who have developed CIN after interventional procedures. Measurement of EPC levels might be useful for screening high CIN risk population before undergoing percutaneous interventions.

CIN, characterized by the development of acute renal failure after exposure to radiocontrast agents, is a common cause of hospital-acquired acute renal injury [17]. Although CIN is generally benign in most instances, it is associated with extended length of hospital stays, increased health care costs, and higher risk of death [4]. As well as an increased risk of death, contrast-induced acute kidney injury is also associated with other adverse outcomes including late cardiovascular events after percutaneous interventions. CIN is generally defined as an increase in serum creatinine concentration of >0.5 mg/dL or 25% above baseline within 48 hours after contrast administration [3]. The risk factors that may predispose patients to CIN after cardiovascular interventional procedures include advanced age, diabetes mellitus, dehydration, and pre-existing renal disease. Several strategies, including volume expansion, using iso-osmolar contrast, and limiting the amount of administered contrast media, have become well established methods for prevention of CIN.

Although the exact mechanisms of CIN have yet to be fully elucidated, several causes have been described. Most likely, a combination of various mechanisms is responsible for the development of CIN. A reduction in renal perfusion caused by a direct effect of contrast media on the kidney, and toxic effects on the tubular cells are generally accepted as the main factors in the pathophysiology of CIN. Accumulating evidence suggests that the acute renal failure caused by the radiocontrast agents seems to be a consequence of an imbalance between vasoconstrictor factors and vasodilator agents like the prostaglandins or NO [18]. The role of NO in renal hemodynamics regulation has been reported in many studies. A decreased NO synthesis, or a lack of response of the endothelium to vasodilators, have been suggested as possible mechanisms for the ischemic or the nephrotoxic ARF [19], [20]. Our study is consistent with previous reports showing that decreased NO concentrations may predispose to CIN after percutaneous interventions. Schwartz *et al.* observed that the administration of radiocontrast agents to rats resulted in a significant decrease in urinary guanosine 3′,5′-cyclic monophosphate, as well as NO_2_^−^ and NO_3_^−^ excretion, and this decrease was significantly attenuated by administration of L-arginine [21]. These results indicate that NO plays a major role in the pathogenesis of acute renal failure induced by radiocontrast agents.

Convincing evidence suggests that atherosclerosis is associated with endothelial dysfunction at the early stage of the disease process. Intact endothelium and maintenance of endothelial integrity play a pivotal role in preventing the development of atherosclerotic vascular disease. Recent insight suggests that the injured endothelial monolayer is regenerated by bone marrow-derived EPC, and circulating EPCs correlate with important clinical outcomes in vascular health [13]. They contribute to angiogenesis and organ repair in both animal and human models of ischemic injury [22], [23]. With regard to renal injury, they appear to home in on, and incorporate into sites of active neovascularization in the kidney [7], [24]. Pastchan *et al*. have demonstrated that, in mice models, renal ischemia rapidly mobilizes EPCs, which transiently home in on the spleen and subsequently accumulate in the medullopapillary region of the kidney [25]. They also proved that EPC-enriched cells from the medullopapillary parenchyma afforded partial renoprotection after renal ischemia, implying an important role of the recruited EPCs in the functional rescue of renal ischemia. It appears that bone marrow-derived EPCs may play a critical role in improving kidney function after ischemic or nephrotoxic injury in experimental models.

EPCs represent a very minor cell population in whole blood, and the choice of markers and controls is very important. However, there is still confusion about the definition used for EPC, and the circulating putative EPC identified in this study may include a monocyte subpopulation that may well have pro-angiogenic properties. However, in attempting an identification of EPC, a major limiting factor is that no simple definition of EPC exists at the present time, while various methods to define EPC have been reported. Therefore, we used CD34^+^, CD34^+^KDR^+^, CD34^+^KDR^+^CD133^+^ markers to identify circulating EPCs in the current study [26]. Our data showed reduced circulating EPC levels were associated with development of CIN, and subsequent cardiovascular events after percutaneous interventions. Recent evidence indicates that mobilization and differentiation of EPCs are modified by NO, and that bone marrow-expressed eNOS is essential for the mobilization of stem cells and progenitor cells in vivo [27]. Therefore, decreased NO concentrations in CIN patients may modulate EPC behaviors and result in impaired vascular repair capacity, which suggests a pivotal role of EPC in modulation of CIN, and that a reduced number of these cells gives rise to the poor prognosis in CIN patients. These findings further provide pathophysiological insights into CIN development and raise the possibility that circulating EPCs may be a target for preventive interventions in selected individuals.

Some limitations of this study should be addressed. First, the sample size of this study was relatively small and may limit the interpretation of the study result. Due to the limited number of CIN patients, we were only able to adjust for 2 covariates in multivariate analysis to avoid over-fitting the problem. To draw a more definite conclusion, a larger population and longer follow-up duration would be required. Second, the EPC results showed relatively large standard deviations; however, these are not unusual for this kind of study. Third, we did not evaluate EPC function or clinical endothelial functions, such as adhesion, proliferation, migratory ability, and endothelium-dependent flow-mediated dilatation. However, we did check the nitric oxide levels in study subjects. Furthermore, a previous study has shown that EPC and endothelial functions exhibited changes in a similar pattern with respect to EPC number [28]. Finally, we did not recheck EPC levels after development of CIN in study subjects and had no idea if there was any distinct pattern of EPC mobilization in CIN patients.

In conclusion, circulating EPCs are decreased in patients who develop CIN, and a reduced number of circulating EPCs is significantly associated with MACE in CIN patients. Our findings may partially explain the pathophysiology of CIN and the poor prognosis in CIN patients. Furthermore, measurement of EPC number might be useful in identifying high CIN risk and high cardiovascular risk population.

## References

[1] Parfrey PS, Griffiths SM, Barrett BJ, Paul MD, Genge M, et al (1989) Contrast material-induced renal failure in patients with diabetes mellitus, renal insufficiency, or both. A prospective controlled study. N Engl J Med 320: 143–149. 2643041 10.1056/NEJM198901193200303

[2] Rihal CS, Textor SC, Grill DE, Berger PB, Ting HH, et al (2002) Incidence and prognostic importance of acute renal failure after percutaneous coronary intervention. Circulation 105: 2259–2264. 12010907 10.1161/01.cir.0000016043.87291.33

[3] McCullough PA, Sandberg KR (2003) Epidemiology of contrast-induced nephropathy. Rev Cardiovasc Med 4 [Suppl 5]: S3–9. 14668704

[4] Tepel M, Aspelin P, Lameire N (2006) Contrast-induced nephropathy: a clinical and evidence-based approach. Circulation 113: 1799–1806. 16606801 10.1161/CIRCULATIONAHA.105.595090

[5] Heyman SN, Rosen S, Khamaisi M, Idee JM, Rosenberger C (2010) Reactive oxygen species and the pathogenesis of radiocontrast-induced nephropathy. Invest Radiol 45: 188–195. 20195159 10.1097/RLI.0b013e3181d2eed8

[6] Wong PC, Li Z, Guo J, Zhang A (2012) Pathophysiology of contrast-induced nephropathy. Int J Cardiol 158: 186–192. 21784541 10.1016/j.ijcard.2011.06.115

[7] Rehman J, Li J, Orschell CM, March KL (2003) Peripheral blood “endothelial progenitor cells” are derived from monocyte/macrophages and secrete angiogenic growth factors. Circulation 107: 1164–1169. 12615796 10.1161/01.cir.0000058702.69484.a0

[8] Bahlmann FH, DeGroot K, Duckert T, Niemczyk E, Bahlmann E, et al (2003) Endothelial progenitor cell proliferation and differentiation is regulated by erythropoietin. Kidney Int 64: 1648–1652. 14531796 10.1046/j.1523-1755.2003.00279.x

[9] Jie KE, Zaikova MA, Bergevoet MW, Westerweel PE, Rastmanesh M, et al (2010) Progenitor cells and vascular function are impaired in patients with chronic kidney disease. Nephrol Dial Transplant 25: 1875–1882. 20083473 10.1093/ndt/gfp749

[10] Perticone F, Maio R, Perticone M, Sciacqua A, Shehaj E, et al (2010) Endothelial dysfunction and subsequent decline in glomerular filtration rate in hypertensive patients. Circulation 122: 379–384. 20625109 10.1161/CIRCULATIONAHA.110.940932

[11] Chu K, Jung KH, Lee ST, Park HK, Sinn DI, et al (2008) Circulating endothelial progenitor cells as a new marker of endothelial dysfunction or repair in acute stroke. Stroke 2008 39: 1441–1447. 10.1161/STROKEAHA.107.499236 18356550

[12] Yip HK, Chang LT, Chang WN, Lu CH, Liou CW, et al (2008) Level and value of circulating endothelial progenitor cells in patients after acute ischemic stroke. Stroke 39: 69–74. 18063830 10.1161/STROKEAHA.107.489401

[13] Werner N, Kosiol S, Schiegl T, Ahlers P, Walenta K, et al (2005) Circulating endothelial progenitor cells and cardiovascular outcomes. N Engl J Med 353: 999–1007. 16148285 10.1056/NEJMoa043814

[14] Levey AS, Bosch JP, Lewis JB, Greene T, Rogers N, et al (1999) A more accurate method to estimate glomerular filtration rate from serum creatinine: a new prediction equation. Modification of Diet in Renal Disease Study Group. Ann Intern Med 130: 461–470. 10075613 10.7326/0003-4819-130-6-199903160-00002

[15] Eknoya G, Levin N (2002) K/DOQI clinical practice guidelines for chronic kidney disease: evaluation, classification, and stratification. Am J Kidney Dis. 39: S1–266. 11904577

[16] Chiang CH, Huang PH, Chung FP, Chen ZY, Leu HB, et al (2012) Decreased circulating endothelial progenitor cell levels and function in patients with nonalcoholic fatty liver disease. PLoS One 7: e31799. 22359630 10.1371/journal.pone.0031799 PMC3280999

[17] McCullough PA (2008) Contrast-induced acute kidney injury. J Am Coll Cardiol 51: 1419–1428. 18402894 10.1016/j.jacc.2007.12.035

[18] Vallance P, Chan N (2001) Endothelial function and nitric oxide: clinical relevance. Heart 85: 342–350. 11179281 10.1136/heart.85.3.342 PMC1729645

[19] Agmon Y, Peleg H, Greenfeld Z, Rosen S, Brezis M (1994) Nitric oxide and prostanoids protect the renal outer medulla from radiocontrast toxicity in the rat. J Clin Invest 1994 94: 1069–1075. 10.1172/JCI117421 PMC295165 8083347

[20] Ribeiro L, de Assuncao e Silva F, Kurihara RS, Schor N, Mieko E, et al (2004) Evaluation of the nitric oxide production in rat renal artery smooth muscle cells culture exposed to radiocontrast agents. Kidney Int. 65: 589–596. 10.1111/j.1523-1755.2004.00408.x 14717929

[21] Schwartz D, Blum M, Peer G, Wollman Y, Maree A, et al (1994) Role of nitric oxide (EDRF) in radiocontrast acute renal failure in rats. Am J Physiol 267: F374–379. 8092251 10.1152/ajprenal.1994.267.3.F374

[22] Zhang ZG, Zhang L, Jiang Q, Chopp M (2002) Bone marrow-derived endothelial progenitor cells participate in cerebral neovascularization after focal cerebral ischemia in the adult mouse. Circ Res 90: 284–288. 11861416 10.1161/hh0302.104460

[23] Tateishi-Yuyama E, Matsubara H, Murohara T (2002) Therapeutic angiogenesis for patients with limb ischaemia by autologous transplantation of bone-marrow cells: a pilot study and a randomised controlled trial. Lancet 360: 427–435. 12241713 10.1016/S0140-6736(02)09670-8

[24] Ricardo SD, Deane JA (2005) Adult stem cells in renal injury and repair. Nephrology (Carlton). 10: 276–282. 10.1111/j.1440-1797.2005.00373.x 15958042

[25] Patschan D, Krupincza K, Patschan S, Zhang Z, Hamby C, et al (2006) Dynamics of mobilization and homing of endothelial progenitor cells after acute renal ischemia: modulation by ischemic preconditioning. Am J Physiol Renal Physiol 291: F176–185. 16478972 10.1152/ajprenal.00454.2005

[26] Iwami Y, Masuda H, Asahara T (2004) Endothelial progenitor cells: past, state of the art, and future. J Cell Mol Med 8: 488–97. 15601577 10.1111/j.1582-4934.2004.tb00473.x PMC6740132

[27] Ozuyaman B, Ebner P, Niesler U, Ziemann J, Kleinbongard P, et al (2005) Nitric oxide differentially regulates proliferation and mobilization of endothelial progenitor cells but not of hematopoietic stem cells. Thromb Haemost 94: 770–772. 16270628 10.1160/TH05-01-0038

[28] Jialal I, Devaraj S, Singh U, Huet BA (2010) Decreased number and impaired functionality of endothelial progenitor cells in subjects with metabolic syndrome: implications for increased cardiovascular risk. Atherosclerosis 211: 297–302. 20171637 10.1016/j.atherosclerosis.2010.01.036 PMC2902610

